# Upstream *SOX9* deletion in a 46,XY girl with acampomelic campomelic dysplasia and absent minipuberty

**DOI:** 10.1186/s13023-025-04125-0

**Published:** 2025-11-22

**Authors:** Anna Szoszkiewicz, Ewelina Bukowska-Olech, Paweł Kurzawa, Anna Sowińska-Seidler, Marek Niedziela, Zofia Kolesińska, Aleksander Jamsheer

**Affiliations:** 1 Department of Medical Genetics, Doctoral School, Poznan University of Medical Sciences, Poznan, Poland; 2 Department of Medical Genetics, Poznan University of Medical Sciences, Poznan, Poland; 3 Department of Laboratory Diagnostics, Poznan University of Medical Sciences, Poznan, Poland; 4 Department of Clinical Pathology and Immunology, Poznan University of Medical Sciences, Poznan, Poland; 5 Department of Pediatric Endocrinology and Rheumatology, Institute of Pediatrics, Poznan University of Medical Sciences, Poznan, Poland; 6 Department of Oncological Pathology, University Hospital in Poznan, Poznan, Poland; 7 Diagnostyka GENESIS, Poznan, Poland

**Keywords:** 17q24 deletion, Position effect, Campomelic dysplasia, *SOX9* regulatory element, Scoliosis, Minipuberty

## Abstract

**Background:**

Campomelic dysplasia (CD) is a rare congenital skeletal dysplasia frequently associated with differences of sex development (DSD). In about 10% of affected individuals, the bowing of the long bones (campomelia) is absent, referred to as acampomelic campomelic dysplasia (ACD). Most patients with ACD carry heterozygous pathogenic variants within the *SOX9* coding region or balanced chromosomal rearrangements involving the 17q24 region. A rarer mechanism involves deletions located upstream of the *SOX9* gene. Only five ACD cases with upstream deletions of *SOX9* have been reported in the medical literature.

**Results:**

We report a female patient affected by ACD with Pierre Robin sequence, complete gonadal dysgenesis (CGD), and hypotonia. Genetic testing revealed a *de novo* 1.671 Mb deletion located 191 kb upstream of the *SOX9* gene. This chromosomal aberration represents the second-largest deletion upstream of *SOX9* reported to date. In addition, we describe the patient’s endocrine profile, which revealed an absent gonadotropin rise during minipuberty, followed by a delayed increase in infancy.

**Conclusions:**

This study expands the clinical and molecular spectrum of ACD, enhancing our understanding of genotype-phenotype correlations of this condition. The phenotypic and endocrinological description of the proband may be helpful for clinicians who consult patients with DSD and skeletal dysplasia.

**Supplementary information:**

The online version contains supplementary material available at 10.1186/s13023-025-04125-0.

## Introduction

Campomelic dysplasia (CD) (OMIM: 608160) is an ultrarare, severe developmental skeletal disorder characterized by shortened and bowed (campomelia) femora and tibiae, hypoplasia of the pelvis and scapular bones, a small chest with missing ribs, cervicothoracic kyphoscoliosis, absent pedicles of the thoracic spine, laryngo-tracheomalacia, club feet, and Pierre Robin sequence (PRS; micrognathia, cleft palate and glossoptosis). Extra-skeletal phenotypes include facial dysmorphism with relative macrocephaly, various brain anomalies (e.g., short callosum, diminished white matter, hydrocephalus, agenesis of olfactory bulbs), as well as congenital heart defects (e.g., tetralogy of Fallot, valvular cardiopathy), and kidney malformations (e.g., hydronephrosis) [1–5]. In about two-thirds of the affected 46,XY individuals, gonadal dysgenesis occurs, resulting in atypical external genitalia in partial forms or complete sex reversal with female external genitalia [5]. Approximately 10% of patients lack campomelia and are classified as acampomelic CD (ACD). Most infants with CD die in the neonatal period due to hypoplastic thorax leading to respiratory distress syndrome; however, patients with ACD typically have a milder disease course, with 65% surviving beyond 1 year of age [5]. Among long-term survivors, cervical spine instability with spine compression, progressive scoliosis leading to compromised lung function, short stature, and hearing impairment are frequently reported.

Most CD and ACD cases result from intragenic heterozygous *de novo* pathogenic variants in *SOX9*. The remaining cases are due to chromosomal rearrangements such as deletions encompassing *SOX9*, reciprocal translocations, deletions, and inversions with breakpoints outside the coding region of the *SOX9* gene [6]. The distance between the chromosomal breakpoint and the *SOX9* gene correlates with the severity of CD. Specifically, the closer a chromosomal rearrangement is to *SOX9*, the more severe the clinical presentation. Over twenty cases of ACD have been reported in the literature. Twelve patients carried missense pathogenic variants in *SOX9*, while sixteen affected individuals had chromosomal rearrangements upstream of *SOX9* [6–30].

In this paper, we describe a patient with ACD, PRS, 46,XY complete gonadal dysgenesis (CGD), and hypotonia resulting from *de novo* ~1.7 Mb deletion of chromosome 17q24.3. We localized the chromosome 17 breakpoint to a region 191 kb upstream of *SOX9*. Our report documents the second-largest deletion near the *SOX9* gene associated with this condition. Additionally, this paper discusses genotype-phenotype correlations in ACD, highlighting optimal management strategies, potential pitfalls associated with absent minipuberty, and considerations for germ cell cancer (GCC) management.

## Materials and methods

### Subjects and DNA extraction

We extracted the genomic DNA (gDNA) of the index patient and her parents from the peripheral blood leukocytes with the MagCore® HF16 Automated Nucleic Acid Extractor (RBC Bioscience Corp.). gDNA was quantified using the Agilent 2200 TapeStation system (Agilent Technologies) and NanoDrop 2000 Spectrophotometer (ThermoFisher Scientific). This study was approved by the Institutional Review Board of the Poznan University of Medical Sciences ethics committee no.: 686/22. Written informed consent was obtained from the patient’s parents.

### Array comparative genomic hybridization (array CGH)

Array comparative genomic hybridization (aCGH) was performed using SurePrint G3 Human CGH Microarray 1 × 1 M (Agilent Technologies) with a median probe spacing of 2.1 kb following the producer’s protocol. Hybridization signals were detected with SureScan Dx Microarray Scanner (Agilent Technologies) and visualized with Agilent CytoGenomics 5.0.2.5 software (Agilent™, Agilent Technologies, Santa Clara, CA) as described previously [31].

### Quantitative real-time polymerase chain reaction (qPCR)

We performed a quantitative real-time PCR (qPCR) to confirm array CGH results and narrow down its genomic coordinates in the patient and her parents. We used the SYBR dye-based master mix (SYBR Green PCR Master Mix; ThermoFisher Scientific) and ran reactions in triplicate on the ViiA™ 7 Real-Time thermal cycler (Applied Biosystems). We applied the comparative 2^−ΔΔCt^ method using healthy control DNA as a calibrator. The results were normalized to the albumin gene (*ALB*) and we performed sex determination of samples targeting the factor VIII gene (*F8*) located on chromosome X. Primers were designed using Primer 3 software. Primer sequences are listed in Supplementary Materials (Table S1).

### Breakpoint sequencing

We identified the exact breakpoints of the aberration using polymerase chain reaction (PCR). Specific primers were designed to amplify the DNA fragment spanning the 3′ and 5′ ends of the deletion at chromosome 17q24.3. The PCR products were sequenced on Applied Biosystems Prism 3700 DNA Analyzer (Applied Biosystems) using dye-terminator chemistry (kit v.3, ABI 3130XL). Sequencing data were analyzed by BioEdit software with alignment to the reference human genome (GRCh38/hg38). The reaction conditions are available upon request. Primer sequences are listed in Supplementary Materials (Table S2).

## Results

### Clinical report

**Fig. 1:**
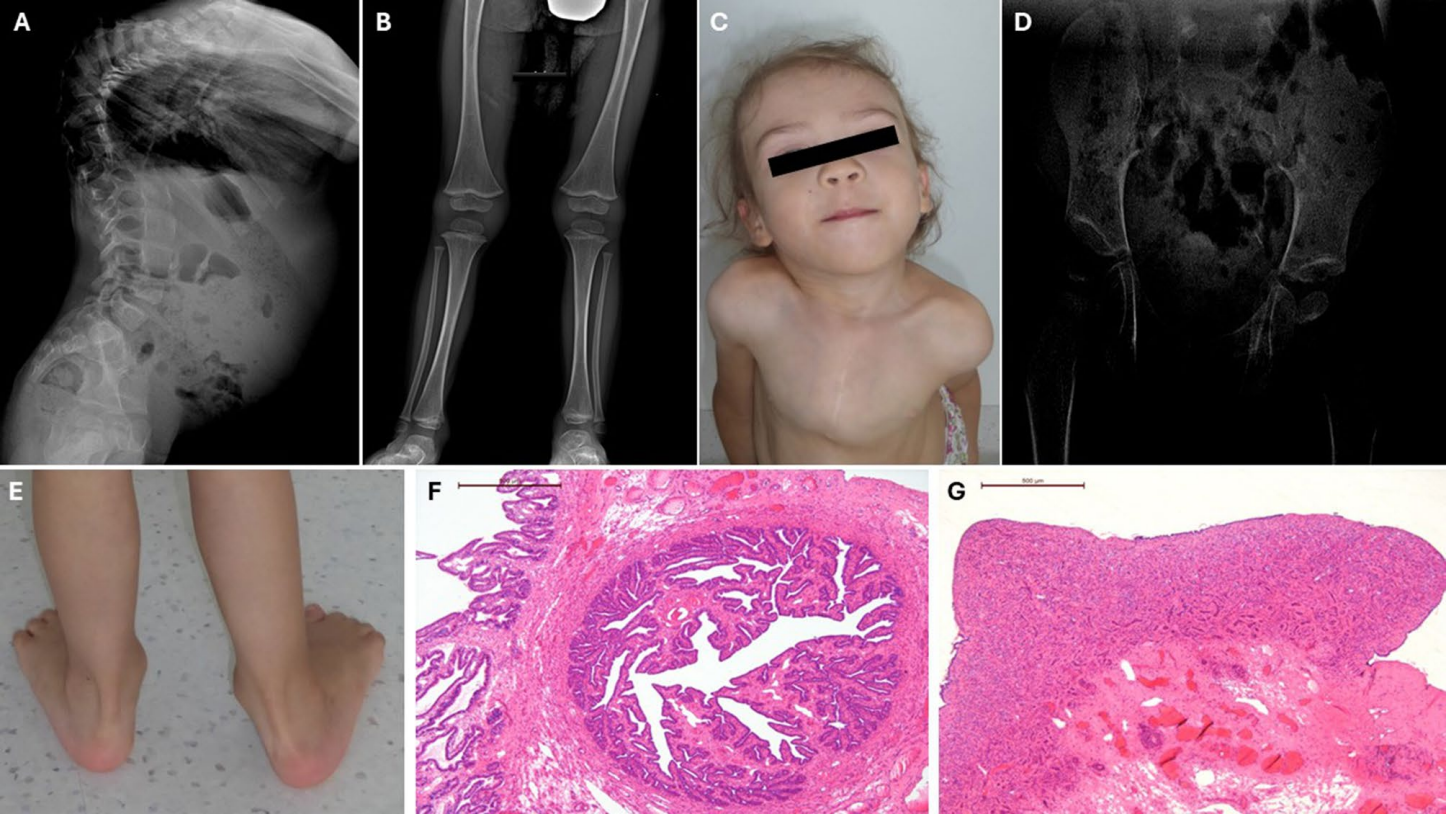
Clinical and radiological features of the index patient. (**A**) Chest radiograph showing severe thoracic kyphoscoliosis with undermineralized thoracic pedicles (age: 15 months). (**B**) Lower extremities X‑ray imaging showing straight femur, tibia, and fibula (age: 15 months) (**C**) Clinical photograph showing short neck with cervical kyphoscoliosis and mild facial dysmorphosism, including long philtrum and low-set ears (age: 3 years 3 months). (**D**) Radiograph of pelvis documenting narrow iliac wings, widening of the pubic bone, and pubic symphysis diastasis (age: 3 years 3 months). (**E**) Photograph of flat-valgus feet (age: 3 years 3 months). (**F**) Cross-section of the fallopian tube with part of the infundibulum on the left. H&E, 100x (age at tissue sampling: 3 years 10 months). (**G**) Section of a streak gonad resembling ovarian stroma. (**H&E**), 100x (age at tissue sampling: 3 years 10 months)

A female infant was born to non-consanguineous parents (a 28-year-old mother and a 29-year-old father) following an uncomplicated 1^st^ pregnancy. She was delivered spontaneously at 41 weeks of gestation, with a birth weight of 2960 g (3-10th percentile), length of 50 cm (50th–75th percentile), head circumference of 34 cm (50th–75th percentile), and Apgar scores of 9 at both 1 and 5 minutes. The mother had a history of hypothalamic amenorrhea prior to pregnancy. After birth, the infant developed respiratory distress necessitating non-invasive respiratory support. Clinical examination revealed a flat midface, cleft soft palate, and small gestational age (SGA) status. On the 3rd day of life, laryngotracheoscopy revealed tracheomalacia over two-thirds of the tracheal length. Computed tomography (CT) angiography identified a vascular ring formed by the brachiocephalic trunk, compressing the trachea and left bronchial lumen. It also raised suspicion of a congenital spinal malformation due to the incomplete closure of the neural tube at the levels of C5–C7 and the thoracic spine. Because of persistent respiratory compromise, the infant underwent a cardiosurgical procedure to suspend the brachiocephalic trunk and remove the right lobe of the thymus. Postoperatively, she experienced acute respiratory distress with apnea and pallor, which resolved with pulmonary rehabilitation. Given multiple associated conditions, she was referred to the clinical genetic department for evaluation. Examination revealed mild dysmorphic features, including a sloping forehead, low-set ears, micrognathia, a plantar fascial groove, a broad nasal bridge, a cleft palate, and a long philtrum; a short neck with a low hairline at the back, widely spaced nipples, a sandal gap, and female external genitalia (Fig. 1). First-line genetic testing confirmed a 46,XY karyotype, prompting an endocrine evaluation. At 2 months of life, a pelvic ultrasound identified a normal-sized uterus but did not visualize the ovaries, leading to a diagnosis of 46,XY CGD. Unexpectedly, although the hormonal assessment was performed twice during minipuberty (at the age of 5 and 8 weeks), gonadotropins were low, accompanied by low levels of estradiol, testosterone, inhibin B, and AMH (Table 1). The serum concentrations of TSH, ACTH, and IGF-1 were within the normal range for age. However, subsequent follow-up visits revealed an increase in gonadotropin levels, consistent with hypergonadotropic hypogonadism typical of CDG. Given the high risk of germ cell cancer in 46,XY CGD, tumour markers were periodically evaluated until a gonadectomy could be performed. Cleft palate repair was completed at 10 months of age. An X-ray examination at 15 months demonstrated multiple skeletal abnormalities, including a short neck, severe thoracic kyphoscoliosis, a bell-shaped narrow thorax, hypoplastic scapulae, poor mineralization of thoracic pedicles, and narrow iliac wings. The thoracic cage deformity precluded accurate assessment of the rib count and straight long bones. At 24 months, a single occurrence of elevated βhCG level was detected (Table 1), prompting bilateral gonadectomy. Of note, the histopathologic evaluation revealed no embryonic tumor or other malignancies (Fig. 1F–G). Over time, the patient developed progressive cervico-thoraco-lumbar kyphoscoliosis, valgus knees, and flat-valgus feet. CT imaging showed a left-directed scoliosis apex at C7 (Cobb angle 77°) and a right-directed apex at Th9-Th10 (Cobb angle 93°), reduced vertebral body height in multiple cervical and thoracic vertebrae, significant vertebral rotation, and spinal canal narrowing at Th1–Th7. Additional findings included hypoplastic transverse processes at C4–C7 on the left, nonunion of vertebral arches at C1, C4, C5, and L3, and lumbar kyphoscoliosis with an L1–L5 Cobb angle of 20° associated with vertebral rotation at L1–L2. Spina bifida was noted from L3 downward, and the lumbar spinal canal was wide (17–18 mm at L1–2) (Fig. 1A). Apart from hypotonia, there were no additional neurological abnormalities. Psychomotor development was slightly delayed in gross motor skills as a consequence of complex spine malformation, muscular hypotonia, and joint hypermobility. Importantly other activities, including crawling, appeared on time, while independent walking was achieved at 17 months. Due to severe malformation of the spine, the patient from the very beginning had shown difficulties in sitting, preferring an upright body position. During the most recent evaluation at the age of 4 years, the patient presented with normal intellectual and social development. She was able to speak entire sentences, although she required electrostimulations of the oral region used for treatment of initially observed speech delay, most probably resulting from palatal surgery and muscular hypotonia.

**Table 1:** Biochemical assessment during minipuberty and infancy

	age						
	reference values	5 weeks	8 weeks	13 months	20 months	24 months	2 years 3 months
LH [IU/L]	0.5–7	0.1	< 0.03	0.67			
FSH [IU/L]	0.5–7	5.4	5.6	38.2 ↑			
E2 [pg/mL]	< 10–30	< 10	< 10				
testosterone [nmol/L]	0.5–12.6	0.24 ↓	0.1 ↓				
Inhibin B [pg/mL]	210–335		< 4 ↓	< 4 ↓			
AMH [ng/mL]	74–290	0.01 ↓	0.01 ↓	0.01 ↓			
AFP [ng/mL]	< 2				< 2	2.78	
βhCG [IU/mL]	< 5				< 2,3	21.84 ↑	< 2,3
LDH [IU/mL]	110–295				301	314	

## Molecular analyses

**Fig. 2:**
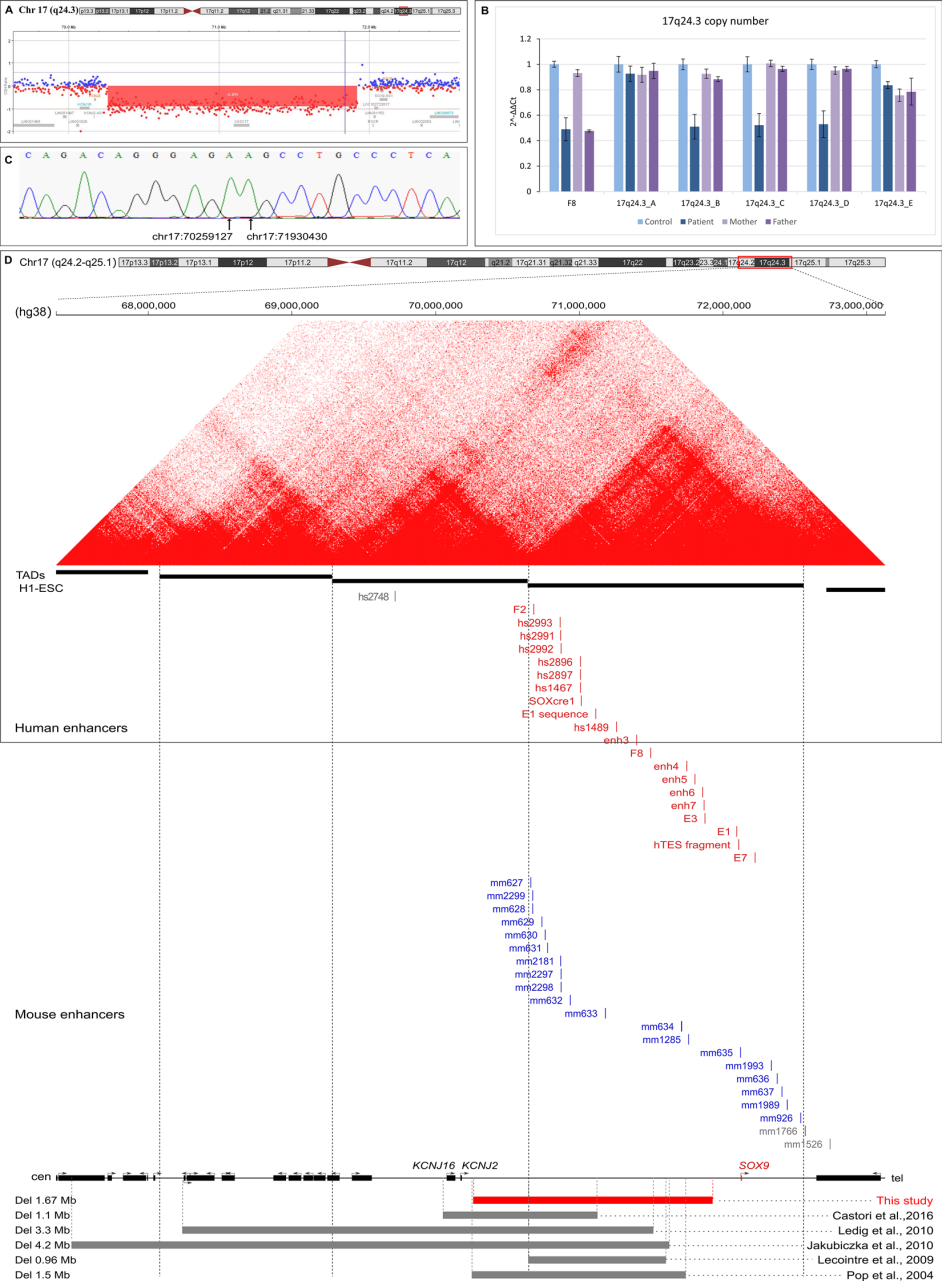
17q24.3 deletion identified in the index patient. (**A**) aCGH results showing a heterozygous 17q24.3 1.671 Mb deletion in the index patient. (**B**) qPCR validation of the 17q24.3 locus in the affected family showing the de novo occurrence of the heterozygous deletion (~0.5 Fold decrease) in the index patient. Error bars represent standard deviation. (**C**) Results of Sanger sequencing of the 17q24.3 deletions’ breakpoints. Black arrows indicate the nucleotide coordinates. The reference sequence was obtained from the UCSC genome browser on human (GRCh38/hg38). (**D**) Mapping of the 17q24.2-q25.1 locus. Upper panel: micro-C chromatin structure on H1-hESC of the locus (hg38: chr17:67,355,513– 73,127,590) [32]. TADs and tad boundaries identified in H1-ESC (H1-ESC_Dixon_2015-raw_tads) [33] are represented by black horizontal bars and vertical black dashed lines respectively. Human and mouse *SOX9* enhancers located within the *SOX9* TAD are indicated by vertical red and blue lines respectively. Enhancers outside the *SOX9* TAD are indicated in gray [34–42]. Bottom panel: horizontal bars and vertical dashed lines indicate deleted regions in the index female patient affected with ACD and 46,XY CGD (red) and previously described (grey): a familial case of five relatives with isolated PRS and ACD with or without congenital heart defect and intellectual disability [29]; a female patient with ACD and 46,XY CGD [30]; a female patient with ACD, PRS, 46,XY sex reversal, muscle hypotonia, double left kidney, and hearing loss [27]; a familial case of a female patient with ACD, PRS, and 46,XY CGD and her mother affected with ACD [6]; a female patient with ACD, PRS, and 46,XY CGD [26]

High-resolution array CGH identified an interstitial deletion at chromosome 17q24.3, with a minimal size of 1.659 Mb (arr[GRCh38] 17q24.3 (chr17:70260910_71919954)x1). The deleted region encompassed one non-protein-coding gene (Fig. 2A). qPCR analysis confirmed the 17q24.3 deletion in the patient and excluded its presence in both parents, suggesting *de novo* occurrence. The sex determination analysis by using qPCR showed sex reversal in the affected individual (Fig. 2B). Subsequent rounds of qPCR narrowed down the deletion region, allowing for breakpoint analysis by Sanger sequencing (Fig. 2C). Breakpoint sequencing revealed the exact size of the aberration as 1.671 Mb (chr17:70259128-71930429; hg38). The deletion included eleven known *SOX9* gene enhancers: F2, hs1467, SOX9cre1, E1, enh3, F8, enh4, enh5, enh6, enh7, and E3. The enhancers within the aberration are listed in Table 2.

**Table 2:** Characterization of *SOX9* enhancers located within the *SOX9* topologically associating domain

Enhancer	Organism	Genomic coordinates (hg38)	Distance from *SOX9*	Tissue/cel-type activity	Inside the patient’s deletion	References
mm627	Mouse	chr17:70661708-70662612	− 1.49 Mb	Limb	Yes	[41, 42]
mm2299	Mouse	chr17:70672169-70675020	− 1.45 Mb	Branchial arch, Nose, Limb	Yes	[41, 42]
mm628	Mouse	chr17:70674180-70675051	− 1.45 Mb	Branchial arch, Facial mesenchyme	Yes	[41, 42]
F2	Human	chr17:70679938-70680186	− 1.44 Mb	Craniofacial	Yes	[35]
mm629	Mouse	chr17:70738535-70739801	− 1.38 Mb	ND	Yes	[41, 42]
mm630	Mouse	chr17:70760872-70762335	− 1.36 Mb	ND	Yes	[41, 42]
mm631	Mouse	chr17:70776187-70777524	− 1.34 Mb	ND	Yes	[41, 42]
hs2993	Human	chr17:70865946-70866482	− 1.26 Mb	Branchial arch, Forebrain, Somite, Genital tubercle	Yes	[41, 42]
mm2181	Mouse	chr17:70866032-70866526	− 1.25 Mb	ND	Yes	[41, 42]
hs2991	Human	chr17:70868174-70868645	− 1.25 Mb	Branchial arch, Limb, Neural tube	Yes	[41, 42]
mm2297	Mouse	chr17:70869447-70870220	− 1.25 Mb	ND	Yes	[41, 42]
mm2298	Mouse	chr17:70871136-70872793	− 1.25 Mb	Branchial arch, Nose	Yes	[41, 42]
hs2992	Human	chr17:70871141-70872793	− 1.25 Mb	Branchial arch, Facial mesenchyme, Nose, Limb, Forebrain	Yes	[41, 42]
mm632	Mouse	chr17:70934016-70934978	− 1.19 Mb	ND	Yes	[41, 42]
hs1467	Human	chr17:71003328-71004627	− 1.12 Mb	ND	Yes	[41, 42]
hs2896	Human	chr17:71003869-71004139	− 1.12 Mb	ND	Yes	[41, 42]
hs2897	Human	chr17:71004155-71004416	− 1.12 Mb	ND	Yes	[41, 42]
SOX9cre1	Human	chr17:71011694-71013769	− 1.1 Mb	Chondrocyte	Yes	[36]
E1 sequence	Human	chr17:71111474-71112664	− 1 Mb	Prostate	Yes	[43]
mm633	Mouse	chr17:71180327-71182188	− 939 kb	ND	Yes	[41, 42]
hs1489	Human	chr17:71257148-71258770	− 860 kb	ND	Yes	[41, 42]
enh3	Human	chr17:71398295-71399413	− 720 kb	ND	Yes	[44]
F8	Human	chr17:71492376-71494252	− 600 kb	Sertoli cells	Yes	[38, 39]
mm634	Mouse	chr17:71709331-71713339	− 408 kb	Branchial arch, Limb	Yes	[41, 42]
enh4	Human	chr17:71743448-71743990	− 375 kb	ND	Yes	[44]
mm1285	Mouse	chr17:71756869-71757688	− 363 kb	ND	Yes	[41, 42]
enh5	Human	chr17:71801057-71801776	− 320 kb	ND	Yes	[44]
enh6	Human	chr17:71854678-71855824	− 260 kb	ND	Yes	[44]
enh7	Human	chr17:71865527-71866550	− 250 kb	ND	Yes	[44]
E3	Human	chr17:71870643-71870738	− 245 kb	Inner ear, cranial neural crest	Yes	[34]
E1	Human	chr17:72092646-72093159	− 28 kb	Node, notochord, gut, bronchial epithelium, pancreas	No	[34]
hTES fragment	Human	chr17:72106290-72109375	− 12 kb	Sertoli cells	No	[40]
mm635	Mouse	chr17:72119013-72119855	− 1 kb	Branchial arch	No	[41, 42]
E7	Human	chr17:72219296-72220434	+93 kb	Forebrain (telencephalon), midbrain	No	[34]
mm1993	Mouse	chr17:72335672-72336822	+209 kb	Forebrain, Hindbrain	No	[41, 42]
mm636	Mouse	chr17:72375552-72377231	+214 kb	Branchial arch, Nose, Limb	No	[41, 42]
mm637	Mouse	chr17:72407436-72407761	+281 kb	ND	No	[41, 42]
mm1989	Mouse	chr17:72445125-72447186	+319 kb	Limb	No	[41, 42]
mm926	Mouse	chr17:72539301-72541055	+413 kb	ND	No	[41, 42]

TAD = topologically associating domain, - = upstream, + = downstream, ND = not determined

## Discussion

In this paper, we have described a female patient with ACD, PRS, hypotonia, and 46,XY CGD, resulting from a *de novo* 1.671 Mb deletion at chromosome 17q24.3. The deletion spanned a region located 191 kb to 1.862 Mb upstream of the *SOX9* gene.

The *SOX9* (SRY-related HMG-box gene 9, OMIM: 608160) gene, located on chromosome 17q24.3, encodes a transcription factor critical for reproductive and skeletal development [45]. SOX9 is involved in various processes during embryogenesis, comprising chondrogenesis and testis, skeletal, heart, inner ear, kidney, and neural crest development. Studies on mouse and human cell lines identified tissue-specific enhancers in the genomic region upstream of the *SOX9* gene [34]. Furthermore, the DNA segment upstream of *SOX9* divides into four clusters: a proximal cluster between 50-375 kb, a sex-determining interval RevSex region between 517-595 kb, a distal cluster between 601 and 932 kb, and a PRS cluster between 1.03–1.26 Mb [13, 35]. The chromosomal aberration removing cis-regulatory elements of *SOX9* can lead to gene haploinsufficiency and result in CD/ACD phenotype [13]. The results of our study indicate a positional effect in *SOX9* expression. The deletion detected in our patient comprised eleven known *SOX9* gene enhancers (Table 2). Among them, F2 enhancer disruption contributes to the pathogenesis of the PRS [35]. PRS is a congenital anomaly characterized by cleft palate, micrognathia, glossoptosis, and long philtrum, causing respiratory distress and feeding problems in the neonatal period. Our patient showed clinical features of PRS, including a cleft palate (surgically corrected in the infantile period), micrognathia (surgically corrected in the infantile period), and a long philtrum, which may result from the loss of the F2 enhancer. On the other hand, the F8 enhancer responds synergistically to sex-determining region Y (SRY) and Wilms tumour protein 1 to regulate expression in male reproductive cells. The deletion of the F8 enhancer may contribute to disorders of gonadal differentiation, which in our patient resulted in sex reversal. Conversely, loss of SOX9cre1 may disrupt SHH-dependent *SOX9* regulation, contributing to impaired chondrogenesis and the patient’s vertebral phenotype [36]. Another enhancer, E3, regulates the expression of *SOX9* in the inner ear and cranial neural crest cells [34]. While the deletion of the E3 may have contributed to the patient’s craniofacial dysmorphia, she did not exhibit hearing impairment. The roles of the remaining deleted enhancers in our patient (hs1467, E1 sequence, enh3, enh4, enh5, enh6, enh7) are poorly characterized in the medical literature. We assume that their loss may contribute to the ACD phenotype by reducing *SOX9* expression; however functional studies are needed to confirm our hypothesis. The intact promoter-proximal elements (E1, hsTES, and E7) suggest preserved baseline *SOX9* expression, which could contribute to the relatively mild phenotype.

Copy number variations affect gene regulation by changing the number of regulatory elements and altering the 3D structure of the genome. Disrupted chromatin architecture occurs via the disorganization of topologically associating domains (TADs), which are megabase-sized chromatin domains where regulatory elements have a high frequency of interaction. TADs are insulated by boundary elements that harbor binding sites for zinc finger transcription factor CTCF and the cohesin protein complexes. In humans, the *SOX9* locus shows separation in two major TADs. First TAD includes the *SOX9* gene and the large gene desert with multiple enhancers for *SOX9*, while the other contains the *KCNJ2* and *KCNJ16* genes [46]. Genomic analysis of our patient identified a deletion encompassing two *SOX9* TADs and a boundary element (Fig. 2D). To date, two studies in murine models and human cell lines revealed that disruption of TADs in *SOX9* locus perturb long-range regulatory interactions and contribute to gene misexpression [46, 47]. These findings suggest that, beyond enhancer loss, alterations in higher-order chromatin structure may have contributed to the patient’s phenotype. Further functional studies on our patient should clarify the extent of chromatin reorganization and its potential impact on gene expression and phenotype.

Most cases of ACD result from heterozygous pathogenic variants in the *SOX9* coding region or balanced chromosome rearrangements. Deletions upstream of the *SOX9* gene are a rare cause of ACD. To date, only five deletions upstream of *SOX9* have been reported in patients diagnosed with ACD [6, 26, 27, 29, 30]. Table 3 compares the clinical characteristics of our patient with those of previously documented cases. Fig. 2 illustrates the exact sizes and positions of deletions upstream of *SOX9*. Interestingly, only the deletion of our patient encompassed the proximal cluster containing five known *SOX9* gene enhancers, i.e., enh4, enh5, enh6, enh7, and E3. All deletions, except the one reported by Castori et al. [29], comprised the RevSex determining region consistent with their phenotype. The deletion identified in our patient is the second-largest deletion around the *SOX9* gene reported in ACD. The largest, approximately 4.2 Mb deletion was described in the individual with ACD who also carried an unbalanced translocation between chromosome 7 and chromosome 17 [46,XY,t(7;17)(q33;q24)] [27]. Similarly to our patient, three previously reported deletions occurred *de novo* [26, 27, 30]. In contrast, two studies reported familial cases of ACD [6, 29]. The infant described by Lecointre et al. shared many common clinical features with our patient, such as facial dysmorphia, sandal gap, thoracic spinal anomaly, scoliosis, normal uterus with no detectable gonads, proper psychomotor development, and onset of independent walking at 17 months.

**Table 3:** Clinical features of the index patient and previously reported cases with upstream deletions of the *SOX9* gene

	index patient	III.1 [29]	II.1 [29]	II.2 [29]	II.3 [29]	I.1 [29]	P12 [30]	P [27]	P [6]	mother [6]	P1 [26]	P2 [26]
CVMs	+		+	+	+		+	+	+		+	+
hypoplastic scapulae	+		+	+					+		+	+
thorax (bell-shaped/narrow)	+		+	+	+						+	
pelvic anomalies	+		+	+	+				+	+	+	+
midface hypoplasia	+	+						+	+		+	+
features of PRS	+	+	+	+	+	+		+	+	+	+	+
hearing impairment			+					+				
microcephaly			+			+						
macrocephaly											+	+
short stature	+								+		+	
underweight	+	+									+	
intellectual disability				+	+							
seizures				+								
hypotonia	+			+				+				
CD											+	
ACD	+		+	+	+	+	+	+	+	+		+
46,XY CGD	+						+	+	+			+
CHD		+										
gonadoblastoma									+			

PRS - Pierre Robin sequence; CHD - congenital heart defect; CVMs - congenital vertebral malformations

Pathogenic variants in *SOX9* manifest mainly as loss-of-function alleles, whereas chromosomal rearrangements that remove enhancers lead to residual gene activity. Patients with chromosomal aberrations around *SOX9* and missense pathogenic variants usually show a milder phenotype of ACD than individuals with loss-of-function pathogenic variants in *SOX9*. In line with these findings, our patient with a deletion upstream of *SOX9* survived the neonatal period and exhibited a relatively mild phenotype. On the other hand, breakpoints occurring in the proximal cluster correlate with increased phenotypic severity, mainly manifesting as skeletal malformations. Severe cervico-thoraco-lumbar kyphoscoliosis, accompanied by nonunion of vertebral arches, reduced vertebral body height, hypoplastic transverse processes, and spinal canal narrowing was the most prominent phenotypic feature of our patient. Other reported cases of ACD have also exhibited severe congenital vertebral malformations (CVMs) [9, 13, 19, 21, 25–29]. These anomalies included a fusion of cervical and thoracal vertebrae, hemivertebra, progressive kyphoscoliosis and lordosis, hypoplastic scapulae, and malformed thoracic vertebral bodies with non-mineralized pedicles. In contrast to our patient, Castori et al. (2016) reported the most distal upstream deletion relative to *SOX9* among all reported cases (Fig. 2D). The affected family exhibited a phenotypic spectrum ranging from isolated PRS to ACD, with remarkably mild skeletal manifestations. Notably, the index patient showed no CVMs. SOX9 plays a crucial role in chondrogenesis and skeletal development, which may explain the presence of CVMs in patients with ACD. Experimental models show that homozygous loss-of-function mutations in *SOX9* block chondrocyte differentiation. In contrast, heterozygous knockout mice exhibit skeletal abnormalities resembling human campomelic dysplasia and die shortly after birth from respiratory failure [48]. We propose that the deletion of *SOX9* enhancers in our patient led to residual *SOX9* expression, contributing to the observed skeletal phenotype.

Minipuberty represents a critical window of opportunity for promptly diagnosing DSD, including CGD [49]. Under normal circumstances, one would expect elevated gonadotropins during this period, reflecting dysgenetic gonads. However, in our patient, gonadotropin levels measured twice at the peak of hypothalamic-pituitary-gonadal (HPG) axis activation remained low and only increased later in life. One possible reason for this absent minipuberty is severe illness (post-cardiac surgery and post-apnea), which may have disrupted pulsatile GnRH secretion - the most stress-sensitive component of the HPG axis [50]. Notably, the patient’s mother also experienced hypothalamic amenorrhea prior to pregnancy, likely triggered by stress and intense physical activity. It is also intriguing that some patients with CD present with absent olfactory bulbs, a hallmark of Kallmann syndrome - a congenital form of hypogonadotropic hypogonadism with anosmia [1, 5]. This condition arises from impaired embryonic migration of the vomeronasal nerve and GnRH neurons from the nasal region to the brain, ultimately leading to isolated GnRH deficiency and loss of smell. However, the subsequent rise in gonadotropin levels suggests that the patient’s GnRH neurons developed normally.

In cases of 46,XY CGD, patients are at high risk for GCC development, necessitating gonadectomy [51]. Until the procedure is performed, tumor markers should be monitored; however, an increase in these markers typically indicates an already invasive form of GCC. Unexpectedly, during the follow-up of our patient, although an elevated βhCG level was detected, histopathology did not reveal dysgerminoma (an invasive form of GCM) or the precursor lesion, gonadoblastoma. Nonetheless, since dysgenetic gonads lack hormonal and germinative potential, and because cancer surveillance is currently not effective, the decision to proceed with bilateral gonadectomy remains justified.

## Conclusions and future directions

In conclusion, we report a patient with ACD carrying the second-largest reported deletion upstream of *SOX9*. Our study supports the hypothesis that chromosomal aberrations near *SOX9* contribute to a milder ACD phenotype. This case broadens the spectrum of *SOX9*-associated chromosomal aberrations and offers an additional understanding of the genotype-phenotype correlations in ACD. Despite the growing number of reported cases, the precise molecular basis underlying ACD remains unclear. Future studies should focus on chromatin interaction analyses to determine whether chromosomal rearrangements disrupt the 3D organization of the *SOX9* locus, leading to altered gene expression. Additionally, further investigations are needed to define the specific roles of regulatory elements surrounding *SOX9*. A deeper understanding of these mechanisms could improve diagnostic strategies and refine the classification of CD/ACD.

## Electronic supplementary material

Below is the link to the electronic supplementary material.

Supplementary Material 1

## Data Availability

Data generated during this study are included in this published article and its supplementary material.

## Abbreviations

CD: Campomelic dysplasia
PRS: Pierre Robin sequence
ACD: Acampomelic campomelic dysplasia
CGD: Complete gonadal dysgenesis
gDNA: Genomic DNA
aCGH: Array comparative genomic hybridization
qPCR: Quantitative real-time PCR
PCR: Polymerase chain reaction
SGA: Small gestational age
CT: Computed tomography
SOX9: SRY-related HMG-box gene 9
SRY: Sex-determining region Y
TADs: Topologically associating domains
CVMs: Congenital vertebral malformations
HPG: Hypothalamic-pituitary-gonadal
