# Supplementary material for: Upstream SOX9 deletion in a 46,XY girl with acampomelic campomelic dysplasia and absent minipuberty
Source: Orphanet J Rare Dis. 2025 Nov 22;21:41. doi: 10.1186/s13023-025-04125-0 (PMC12874664; doi:10.1186/s13023-025-04125-0)
Supplement: Supplementary file 1 — Supplementary Material 1 [file 13023_2025_4125_MOESM1_ESM.docx]

**Table S1** Oligonucleotide primers used to perform copy number analysis of the 17q24.3 region.

*** -** primers used for mapping the breakpoints

| **Primer name** | **Sequence (5'->3')** | **Genomic coordinates (hg38)** | **Target** | **Copy number** |
| --- | --- | --- | --- | --- |
| **Reference gene** | | | | |
| ALB_F | TGAAATGGCTGACTGCTGTG | chr4:73408650-73408732 | Albumin (*ALB*) | Normal |
| ALB_R | GGAGGTTTGGGTTGTCATCT |  |  |  |
| **Sex determination** | | | | |
| F8_F | TTTCCATTCAACACCTCAGTCGT | chrX:154999491-154999575 | Factor VIII (*F8*) | Deletion |
| F8_R | GCCTTGGCTTAGCGATGTTG |  |  |  |
| **17q24.3 region** | | | | |
| 17q24_AF | GCAGAGAGGCAGACGTAATC | chr17:70260666-70260748 | 17q24.3 | Deletion |
| 17q24_AR | AAGAGGTGTGTTTGAGCTGG |  |  |  |
| 17q24_BF | GCAGGTCTCAGCAAGTACAA | chr17:70271845-70271924 |  |  |
| 17q24_BR | CATTCTGTCCCTGGAGCATC |  |  |  |
| 17q24_CF | CATAGAGCAGCCGAGTCTTC | chr17:71123703-71123791 |  |  |
| 17q24_CR | AACCGCTTCTTGTCTCAGTG |  |  |  |
| 17q24_DF | GAGAGGCTCACTGTGGTCTA | chr17:71846017-71846106 |  |  |
| 17q24_DR | AGTGGAAGGGCTCTGGTTAT |  |  |  |
| 17q24_EF | AAACGGTCATCCAGTCCAAC | chr17:71923330-71923410 |  |  |
| 17q24_ER | TGGGCTGTTTAGGAGGTAGT |  |  |  |
| 17q24_FF | TAGCAGGCTGTGATCCCAA | chr17:70259331-70259419 |  |  |
| 17q24_FR | CTGGTTCTAAAAGGTGGGACA |  |  |  |
| 17q24_GF | GGAGAGCCCGACATGTATAAG | chr17:71925132-71925215 |  |  |
| 17q24_GR | AGCTTGTACTTAATCCTGGCC |  |  |  |
| 17q24_HF | TTAAGTTCAACACCCTGGCA | chr17:70257425-70257504 |  | Normal |
| 17q24_HR | ACTAGTGTGTTCTTGGGCATG |  |  |  |
| 17q24_IF | AATGGGTAGCCTGACTCTCC | chr17:71926737-71926817 |  | Deletion |
| 17q24_IR | GTTGTGAGTGAGTGCATTGCT |  |  |  |
| 17q24_JF | AAGGGGTTGTAGCTGGAGAA | chr17:71929612-71929701 |  |  |
| 17q24_JR | CAGAAACTCAGGCAGGCTCT |  |  |  |
| 17q24_KF | TCTCCAATCCCCAACCTGAA | chr17:70259060-70259139 |  |  |
| 17q24_KR | TTTTCCTCCAGCTCTCCCTG |  |  |  |
| 17q24_LF | **GAACTACCCTTCCCAACCCT*** | chr17:70258470-70258564 |  | Normal |
| 17q24_LR | AAGTGAATGGTCCAGAATCAGTC |  |  |  |
| 17q24_MF | AATGAAGCCGAAGTGTCAGC | chr17:71930031-71930115 |  | Deletion |
| 17q24_MR | GGCATGCTGAACCTTGGAAT |  |  |  |
| 17q24_NF | TGCATATGTGTCCACGAGTG | chr17:71930997-71931082 |  | Normal |
| 17q24_NR | **TCTGACCTAACTTGCCACTG*** |  |  |  |
| 17q24_OF | ACACGATAAAGAGGGAAGAGCT | chr17:71930409-71930480 |  | Deletion |
| 17q24_OR | ACATGAAACAAAGGGCTTCCA |  |  |  |
| 17q24_PF | GTCTTGCTCTGTTGCCCAAA | chr17:71930653-71930752 |  | Normal |
| 17q24_PR | CTGTAGGCTGAAGTGGGAGG |  |  |  |
